# Diet cost and quality using the Healthy Eating Index-2015 in adults from urban and rural areas of Mexico

**DOI:** 10.1017/S1368980021004651

**Published:** 2022-09

**Authors:** Katherine Curi-Quinto, Mishel Unar-Munguía, Sonia Rodríguez-Ramírez, Elin Röös, Walter C Willett, Juan A Rivera

**Affiliations:** 1 Center for Research on Nutrition and Health, National Institute of Public Health, Cuernavaca, Morelos, Mexico; 2 Department of Energy and Technology, Swedish University of Agricultural Sciences, Uppsala, Sweden; 3 Department of Nutrition, Harvard School of Public Health, Boston, MA, USA; 4 National Institute of Public Health, Cuernavaca, Morelos, Mexico

**Keywords:** Diet quality, Diet cost, Drivers of diet quality, Urban and rural, Mexico

## Abstract

**Objective:**

To assess the association between diet cost and quality by place of residence.

**Design:**

We analysed cross-sectional data of the National Health and Nutrition Survey-2012. Diet cost was estimated by linking dietary data, obtained from a 7-d SFFQ, with municipality food prices, which were derived from a national expenditure survey. Diet quality was assessed using the Healthy Eating Index-2015 (HEI-2015). Association between quintiles of diet cost and HEI-2015 was assessed using linear regression analysis.

**Settings:**

Mexico.

**Participants:**

2438 adults (18–59 years).

**Results:**

Diet cost was positively associated with diet quality (HEI-2015) in urban but not in rural areas. Compared with quintile (Q1) of cost, the increment in diet quality score was 1·17 (95 % CI –0·06, 4·33) for Q2, 2·14 (95 % CI –0·06, 4·33) for Q3, 4·70 (95 % CI 2·62, 6·79) for Q4 and 6·34 (95 % CI 4·20, 8·49) for Q5 (*P*-trend < 0·001). Individuals in rural *v*. urban areas on average have higher quality diets at lower cost with higher intakes of whole grains and beans and lower intakes of Na, added sugars and saturated fats. Living in the South, being indigenous and having low socio-economic status were also associated with higher quality diets.

**Conclusions:**

Diet cost was positively associated with diet quality, but only in urban areas. Further studies are needed to understand the relation between diet cost and quality in rural areas. To improve overall diet quality in Mexico, strategies that aim to reduce the cost of high-quality diets should consider the heterogeneity by place of residence.

Globally, current diets do not adhere to dietary guidelines and are considered suboptimal or low-quality diets^(1)^. These types of diets have been associated with the high prevalence of excess weight and non-communicable chronic diseases, as well as with the coexistence of nutritional deficiencies, especially in low- and middle-income countries^(1,2)^. If the quality of diet is not improved, the economic burden associated with treatment costs and productivity losses could reach up to USD 1·3 trillion in 2030 worldwide, disproportionately affecting low-income countries and vulnerable populations that already have less capacity to face health expenditures^(3)^.

Higher prices for healthy food could become a barrier to adopting high-quality diets. This is because food prices are a major driver of food choices that affects food affordability and imposes budgetary restrictions on household purchases^(4)^. This could partly explain the dietary and health disparities observed among vulnerable populations^(5)^. Most of the evidence about the relationship between diet cost and quality comes from high-income countries, showing that high-quality diets are more expensive than low-quality diets^(6)^. However, little is known about this relationship in low- and middle-income countries and vulnerable populations. There is evidence that for rural and indigenous people, high-quality diets can be more affordable than those of low quality^(7–9)^. Also, in some ethnic populations, higher quality diets could be achieved at a lower cost compared with diets followed by the average population^(10,11)^. This variability confirmed the need to better understand the association between the cost and quality of diets in different contexts, which is particularly important in low- and middle-income countries that are more affected by changes in income and food prices than high-income countries^(12)^. Also, low- and middle-income countries are in a rapid but not homogenous process of nutrition transition, associated with socio-economic and cultural characteristics within the country that could hinder the effectiveness of policies intended to improve diet quality in those contexts.

In Mexico – a middle-income country – there is a high prevalence of non-communicable diseases, particularly in the adult population (75·2 % had excess weight, 18·4 % had hypertension and 10·3 % had diabetes in 2018)^(13)^. Most of the evidence about diet quality has been evaluated using *a posteriori* characterisation of food patterns and by assessing adherence to recommendations for nutrients or food groups^(14,15)^. However, few studies have evaluated the overall quality of diet using *a priori* dietary indices, and there is no validated method to measure diet quality such as the Healthy Eating Index-2015 (HEI-2015)^(16)^. This index considers the potential interaction of diet components and assesses diet quality using pre-defined criteria based on dietary recommendations for disease prevention. This allows diet quality to be compared among groups and identify their associated factors^(17,18)^. Also, in a multiethnic cohort from the USA, higher values of the HEI-2015 were associated with a lower risk of all-cause mortality, CVD and cancer in adults^(19)^.

Furthermore, a few studies suggested that less healthier foods are less costly and more affordable than healthier options in Mexico^(20,21)^. Despite this evidence, higher prices on individual healthy food products do not imply that the overall cost of a high-quality diet will be higher because people choose and combine food and beverages of different qualities and prices. Moreover, knowledge is still limited regarding a more comprehensive assessment of the overall quality of the Mexican diet and its relation with diet cost, especially considering differences by place of residence. This is important since differences in food intake and prices have been observed by place of residence; traditional food is more common in rural areas while a ‘western style diet’ is more frequent in urbanised areas^(21)^, and the cost of a basic food basket is higher in urban than in rural areas^(22)^. Therefore, the aim of this study was to assess the association between diet cost and quality among the Mexican adult population (18–59 years) and to examine whether the association varies by place of residence.

## Methods

### Study population and dietary information

This was a cross-sectional study based on the analysis of dietary and socio-demographic data from adult (18–59-year-old) participants of the National Health and Nutrition Survey 2012 (Encuesta Nacional de Salud y Nutrition or ENSANUT-2012 by its Spanish acronym) that was conducted between October 2011 and May 2012. This is a stratified and multi-stage random survey with representativeness at national, state and rural/urban levels^(23)^. The dietary data were obtained from a 7-d semi-quantitative FFQ (SFFQ) collected by trained interviewers using a standardised methodology^(24)^. The SFFQ was validated with a 24-h recall and included 140 food items classified into fourteen groups that contributed to more than 90 % of total energy and nutrient intake^(24)^. The study protocol of ENSANUT was approved by the Ethics Committee of the National Institute of Public Health in Mexico (INSP by its Spanish acronym), and only participants who agreed and signed an informed consent were included in the survey.

### Selection of the analytical sample

Our analytical sample consisted of all adults with complete dietary and socio-demographic data. From an initial sample of 2792 adults (18–59-year-olds) with SFFQ data, we excluded 147 pregnant or lactating women and 207 adults with implausible nutrient intakes (see online supplementary material, Supplemental Fig. 1). We did not consider older adults in the analytical sample (526 individuals > 60 years old) because their dietary intake could be more affected by their physiological condition and potential health problems compared with younger adults^(25,26)^.

The identification of implausible data is fully described elsewhere^(24)^. Briefly, we excluded adults that either reported: (1) intakes in grams of one or more foods above 3 sd, (2) ratios between the intake of energy, macro and micronutrients and their respective required intake above 3 sd and (3) ratios of energy intake to BMR below 0·5.

### Assessment of diet quality

We used the HEI-2015^(27)^, which is a validated method to assess overall diet quality and its individual dietary components by assigning a score according to adherence to the latest Dietary Guidelines for Americans, which reflects international scientific recommendations to promote better food patterns and reduce the risk of non-communicable diseases^(28)^. The HEI-2015 includes thirteen dietary components divided into nine adequacy components: total fruits, whole fruits, total vegetables, greens and beans, whole grains, dairy products, total protein foods, seafood and plant proteins and fatty acids (a ratio of PUFA and MUFA to SFA), for which higher scores represent higher intakes, and four moderation components (refined grains, Na, added sugars and saturated fats), for which higher scores represent lower consumption^(29)^. Each component contributes 0–5 or 0–10 points to the total HEI-2015 score which ranges from 0 to 100 points. In the food grouping, we did not double count the group of greens and beans in the group of total protein and in the group of seafood and plant proteins. Therefore, the first group only included animal food protein and the second group included seafood, seeds and nuts. Details of the food groups and the scoring method are shown in online supplementary material, supplemental Table 1.

To calculate the HEI-2015 for each individual, we followed the procedures described in the National Cancer Institute website^(29)^. Briefly, we calculated the amount of each HEI-2015 dietary component expressed by its equivalent unit, which assesses adherence to dietary recommendations (see online supplementary material, Supplemental Table 1). The equivalent units for food groups were obtained from the ‘Food Patterns Equivalents Database 2013–14’^(30)^. In the case of dishes, we first disaggregated them into ingredients using standardised recipes developed by the INSP. For nutrients (fatty acids, Na, saturated fats), we calculated their total intake based on the INSP’s food composition table. For added sugar, we used the method proposed by Louie *et al.* ^(31)^. In brief, the content of added sugar was zero for foods without sugar or whose sugar is only intrinsic (i.e. fruits); in foods where the main component was added sugar like sweets, the added sugar was equal to the total sugar content. For foods with a mixed content of sugar (intrinsic and added), such as natural juices with sugar, the added sugar was estimated based on standardised recipes developed by the INSP. For processed foods such as yogurt, the content of added sugar was based on the Nutrient Profile Model of the Pan American Health Organization^(32)^.

### Assessment of food prices and diet cost

Our estimation of food prices considered differences between rural and urban areas and was made at the municipal level using data from the National Survey of Household Income and Expenditure 2012 (Encuesta Nacional de Ingresos y Gastos de los Hogares or ENIGH 2012 by its Spanish acronym). This survey has a stratified probabilistic design with national representativeness for urban and rural areas and provides information on the quantity of food and beverages purchased and expenditures per household during the last week. This information is self-reported by the head of the household and includes a list of 220 most consumed food and non-alcoholic beverage items which are considered in the Mexican basic food basket to assess the Consumer Price Index and food poverty lines^(33)^.

We estimated the price per 100 g of each food and beverage by dividing the total monetary expenses for each item by the quantity in grams purchased by household. For milk provided by the national program ‘*Liconsa*’, we used its subsidised price for 2012^(34)^. Then we estimated the median price of each food and beverage at the municipality level. For the sixty-three foods and beverages (28·6 % of items) whose prices at municipality level were missing since no purchases were reported, we assigned the median of food prices at the state level (fifty-eight items) or urban/rural area (six items) when food prices were also missing at the state level. To reduce the potential error in the estimation of prices, we excluded food and beverage items whose quantities and prices were in the first or the ninety-ninth percentile of the distribution. Prices above 2 sd were replaced by the average price for each food item plus 2 sd ^(35)^. All prices were deflated to 2018 using the National Consumer Price Index^(35)^. All of the 161 SFFQ food and beverage items reported in ENSANUT-2012 were matched manually with the most closely related food and beverage item reported in ENIGH, considering their ingredients and nutritional composition. Most of the SFFQ food items were matched with one specific ENIGH item, although some foods such as beef, pork, chicken offal, cheese and mixed sausage were paired with the average of the different cuts or subtypes of meat reported in the ENIGH. Additionally, we converted the quantity of the SFFQ from as-eaten to raw weight using conversion factors from the National Institute of Public Health, since purchased food and beverages are expressed in raw weight in the ENIGH. Food and beverage prices at the municipality level were linked to each individual in ENSANUT-2012 considering their geographical residence. For municipalities in ENSANUT-2012 for which prices were lacking in ENIGH, we assigned the prices of the nearest municipality based on the geographical coordinates for each municipality provided by INEGI-2010, using the Stata module ‘Geonear’ to calculate the geodetic distances between municipalities^(36)^. Finally, the daily diet cost per person was estimated by adding the multiplication of the price by the quantity consumed of each food item and adjusting to 8368 kJ (2000 kcal). For the sensitivity analysis, we used the food prices of INEGI 2012 (National Institute of Statistics and Geography), collected in forty-six urban cities (population above 20 000 habitants), which are used to calculate the Consumer Price Index in Mexico^(35)^.

### Socio-demographic variables

We included sex (male/female), age in tertiles, socio-economic status (SES) in tertiles; education level categorised as low (elementary school or no education), medium (high school) and high (university); ethnicity (indigenous and non-indigenous) and place of residence (urban/rural area and region). The SES was based on an index of household well-being constructed by ENSANUT using principal component analysis of household characteristics, goods and services^(37)^. Ethnicity was categorised as indigenous (when the adult speaks any indigenous language) or non-indigenous^(38)^. Residence area was defined as rural (locations with < 2500 inhabitants) or urban (locations with ≥ 2500 inhabitants)^(39)^, and region of residence was divided into the North, Centre, Mexico City and South.

### Statistical analysis

Descriptive analysis was presented using proportions and means with 95 % CI. We compared the mean of dietary components by the lowest (Q1), middle (Q3) and highest (Q5) quintiles of diet cost using the ANOVA and the Bonferroni *post hoc* analysis for multiple comparison. We stratified the analysis by urban and rural areas if the interaction between area of residence and diet cost was statistically significant (*P*-value < 0·05). To assess the association between quintiles of diet cost and diet quality, we estimated three regression models: (1) crude, (2) adjusted by socio-demographic variables and (3) including the region and the interaction term between cost and area to examine whether the association varied by area of residence.

Sensitivity analysis examined whether the association between diet cost and diet quality could be affected by the source of prices (ENIGH *v*. INEGI prices) as well as the inclusion of the total energy intake as a covariate in the analyses. A *P*-value < 0·05 was considered as statistically significant, and all analyses were conducted in Stata software v.14 using the ‘svy’ prefix for complex surveys. We calculated the adjusted diet quality means using the *margin* command in Stata.

## Results

The means of HEI-2015 and diet cost by socio-demographic characteristics are shown in Table 1. The mean HEI-2015 score of the overall sample was 54·1 (95 % CI 53·5, 54·7), and the mean of diet cost was 52·1 Mexican pesos ($MXN) per 8368 kJ (2000 kcal) (95 % CI 51·1, 53·0). Significant differences in mean quality and cost of diet by SES, education, area, region and ethnicity were observed. When we examined the HEI-2015 of the lowest, middle and highest quintiles of diet cost, adults from rural areas had higher HEI-2015 scores than those in urban ones (Table 2). Furthermore, the mean HEI-2015 scores for fruits, vegetables, dairy products, total protein food, seafood and plant protein, and refined grains were higher among higher quintiles of diet cost in both rural and urban areas (*P* < 0·05). By contrast, the HEI-2015 scores for greens and beans, whole grains, fatty acids (a ratio of PUFA and MUFA to SFA), Na, added sugars and saturated fats were lower among higher quintiles of diet cost (*P* < 0·05) (Table 2).

**Table 1:** HEI-2015 and diet cost among adults (18–59 years) by socio-demographic characteristics, ENSANUT-2012

			HEI-2015§ score		Daily diet cost ($MXN/8368 kJ)	
	*n*	%‡	Mean	95 % CI	Mean	95 % CI
Population						
Total sample	2438	100	54·1	53·5, 54·7	52·1	51·1, 53·0
Sex (%)						
Male	1029	47	53·6	52·7, 54·6	51·1	49·8, 52·4
Female	1409	53	54·5	53·7, 55·3	53·0	51·7, 54·2*
Age group (years)						
18·0–< 29·6	869	33	53·0	51·9, 54·1	52·1	50·8, 53·3
29·6–< 43·5	818	33	54·9	53·9, 55·9*	52·8	51·2, 54·5
43·5–59·0	751	33	54·3	53·2, 55·4	51·3	49·6, 52·9
Education level (%)						
Low	112	4	57·6	54·7, 60·5	47·5	43·2, 51·7
Medium	1561	63	54·2	53·4, 54·9*	49·0	47·9, 50·0
High	765	33	53·5	52·3, 54·6*	58·6	57·0,60·2*,†
Socio-economic level (%)						
Low	818	25	57·8	56·7, 58·9	45·3	44·0, 46·7
Medium	820	33	52·9	51·9, 54·0*	51·3	49·9, 52·7*
High	800	42	52·7	51·8, 53·7*	56·8	55·1,58·5*,†
region (%)						
North	612	21	49·7	48·6, 50·9	55·3	53·3, 57·4
Centre	871	32	55·1	56·6, 58·6*	52·6	50·9, 54·3*
Mexico City	124	17	51·0	51·2, 55·1†	53·3	50·2, 56·5
South	831	31	57·7	59·3, 61·5*,†,||	48·7	47·3, 50·0*,†,||
Residence area (%)						
Urban	1636	77	52·4	51·7, 53·1	54·0	52·8, 55·2
Rural	802	23	59·7	58·6, 60·8*	45·8	44·3, 47·3*
Ethnicity (%)						
Indigenous	216	7	62·2	60·0, 64·5	46·2	43·1, 49·4
Non-indigenous	2222	93	53·5	52·8, 54·1*	52·5	51·5, 53·5*

HEI-2015, Healthy Eating Index-2015.

* Indicates significant difference with the first category.

† Indicates significant difference with the second category.

‡ Percentage of the expanded population size (*n* 51 807 582).

§ Healthy Eating Index-2015. This indicator was used to assess the overall diet quality that ranges from 0 to 100.

|| Indicates significant difference with the third category. The significance was assessed at *P* < 0·05 using a *t* test for mean comparison.

**Table 2:** Mean score of HEI-2015 and dietary components by diet cost tertiles and area of residence, ENSANUT-2012

	Quintiles of diet cost ($MXN/2000 kcal)												
	Urban (mean ± sd)*							Rural (mean ± sd)*					
	Maximum HEI-2015	Lowest (Q1) (*n* 407)		Middle (Q3) (*n* 574)		Highest (Q5) (*n* 655)		Low (Q1) (*n* 404)		Middle (Q3) (*n* 236)		High (Q5) (*n* 162)	
Daily diet cost ($MXN/2000 kcal)		35·0	4·5	51·2	2·1*	73·7	12·1*,†	33·2	5·1	50·6	2·0*	70·2	8·8*,†
HEI-2015 mean score	100	50·2	10·3	51·2	10·3*	55·6	9·5*,†	61·3	12·2	59·1	10·9*	57·8	9·7*,†
Daily energy intake (kcal)		1820	581	1991	692	1707	664†	1797	582	1744	663*	1650	571†
Score for adequacy components													
Total fruits‡	5	2·16	1·71	3·00	1·85*	3·94	1·58*,†	2·23	1·73	3·65	1·44*	3·67	1·80*,†
Whole fruits§	5	3·04	2·04	3·65	1·78*	4·30	1·49*,†	3·15	1·88	4·42	1·25*	4·13	1·48*,†
Total vegetables||	5	1·82	1·07	2·69	1·37*	3·68	1·34*,†	1·98	1·25	2·95	1·38*	3·45	1·55*,†
Greens and beans	5	2·94	1·76	2·48	1·68*	2·27	1·68*	3·56	1·77	2·96	1·87*	3·52	1·73*,†
Whole grains¶	10	4·19	3·92	3·48	3·66	3·10	3·19*	7·40	4·04	4·47	4·20*	5·02	4·15*,†
Dairy products**	10	2·63	2·50	4·01	2·91*	5·48	3·22*,†	2·24	2·63	4·29	2·91*	4·89	3·38*,†
Total protein food††	5	2·00	1·13	3·24	1·31*	3·63	1·38*,†	1·74	1·16	3·01	1·46*	3·41	1·35*,†
Seafood and plant protein‡‡	5	0·45	1·08	0·89	1·41*	1·47	1·76*,†	0·64	1·21	0·96	1·68	1·16	1·61*,†
Fatty acids§§	10	4·20	3·26	3·13	3·07*	2·38	2·55*,†	5·67	3·76	3·72	3·00*	2·37	2·54*,†
Score for moderation components||||													
Refined grains	10	2·18	3·72	3·95	3·37*	6·72	3·29*,†	6·43	4·41	5·87	4·09*	7·97	2·69*,†
Na	10	8·86	2·12	8·45	2·54	7·04	3·17*,†	8·97	2·41	8·44	2·51*	7·05	3·12*,†
Added sugars	10	7·69	2·56	5·80	3·32*	6·52	3·17*	8·39	2·15	7·11	2·92*	5·08	3·16*
Saturated fats	10	8·09	2·49	6·44	3·05*	5·01	3·10*,†	8·94	2·12	7·27	2·42*	6·06	3·71*,†

HEI-2015, Healthy Eating Index-2015.

* Indicates significant difference with the first category.

† Indicates significant difference with the second category. The significance was assessed at *P* < 0·05 using the Bonferroni *post hoc* test of ANOVA for mean comparison of the groups.

‡ Includes 100 % fruit juice.

§ Includes all forms except juice.

|| Includes only vegetables.

¶ Includes maize tortillas, whole bread and cereal bars ≥ 6 g % of fibre.

** Includes all milk products, such as fluid milk, yogurt and cheese.

†† Includes only animal protein food.

‡‡ Includes only seafood and nuts.

§§ Ratio of PUFA and MUFA to SFA.

|||| Moderation components scores of HEI-2015 indicate that higher intakes are related to lower scores and vice versa.

**Fig. 1:**
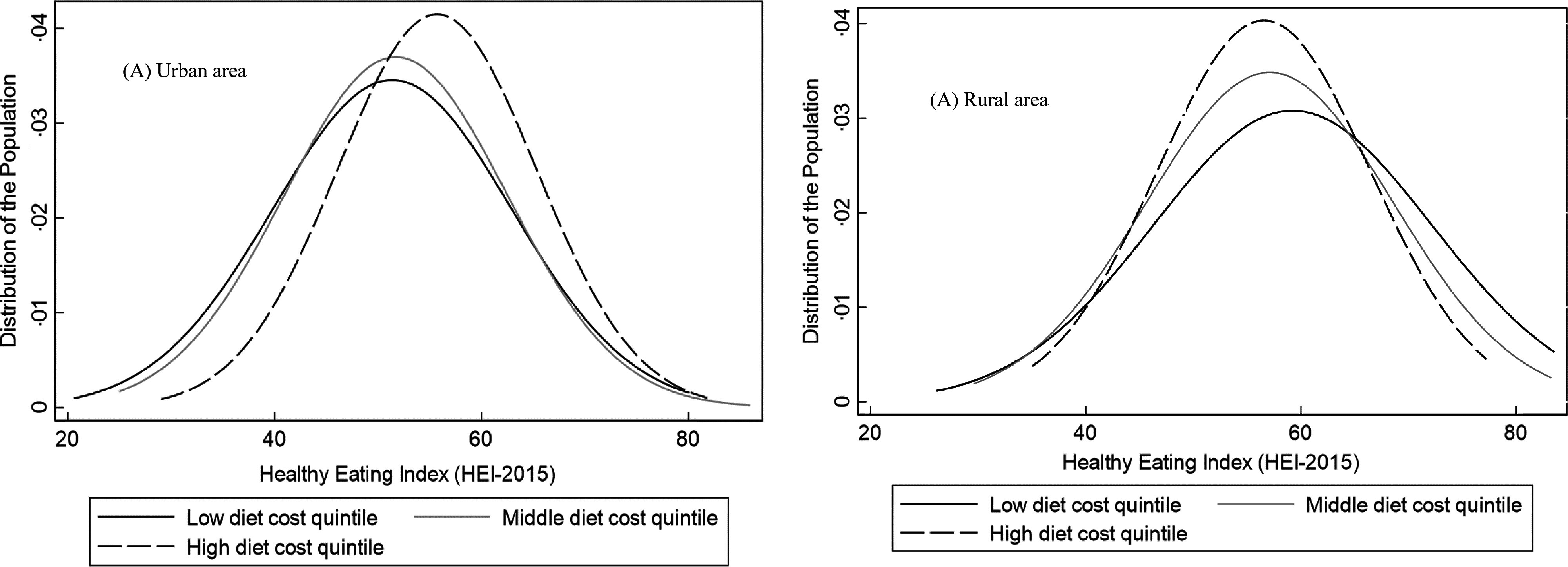
Distribution of Healthy Eating Index-2015* score by low, middle and high quintiles of diet by area of residence. *Diet quality was adjusted by socio-demographic variables using the multiple linear regression model adjusted for sex, age, socio-economic status, education and ethnicity

In the analysis of associations between diet cost and the HEI-2015, we identified a significant interaction between area of residence and diet cost (see online supplementary material, Supplemental Table 2), so we presented the results stratified by area of residence in Table 3. In urban areas, a higher diet cost was associated with a higher HEI-2015 score in quintiles of cost Q3, Q4 and Q5 *v*. Q1, but in rural areas, the association between diet cost and diet quality was not statistically significant for any cost quintile (Table 3). Despite these results, we observed a large area of overlap between the distribution of the HEI-2015 by low, middle and high quintiles of diet cost in both urban and rural areas (Fig. 1).

**Table 3:** Multivariate association between quintiles of diet cost and HEI-2015 in adults (18–59 years) by urban and rural areas, ENSANUT 2012

	Urban (*n* 1636)			Rural (*n* 802)		
	HEI-2015 score			HEI-2015 score		
	Adjusted coefficient	95% CI	*P*-value	Adjusted coefficient	95% CI	*P*-value
Quintiles of diet cost ($MXN/2000 kcal)						
Quintile 1	Reference					
Quintile 2	1·17	–1·01, 3·35	0·293	−2·76	–5·73, 0·21	0·068
Quintile 3	2·14	–0·06, 4·33	0·056	−0·73	–3·79, 2·34	0·641
Quintile 4	4·70	2·62, 6·79	< 0·001	1·75	–2·20, 5·70	0·385
Quintile 5	6·34	4·20, 8·49	< 0·001	−1·39	–4·57, 1·79	0·39
*P*-trend			< 0·001			0·866
Age (years)						
18·0–< 29·6	Reference					
29·6–< 43·5	2·35	0·70, 4·00	0·005	−0·33	–2·64, 1·98	0·777
43·5–59·0	1·13	–0·61, 2·87	0·204	1·72	–0·94, 4·38	0·204
Socio-economic index						
Low	Reference			Reference		
Medium	−1·88	–3·68, –0·07	0·042	−2·22	–4·72, 0·29	0·083
High	−1·03	–2·89, 0·83	0·276	−4·76	–8·15, –1·37	0·006
Sex						
Male	Reference					
Female	0·86	–0·37, 2·09	0·170	−0·64	–2·29, 1·64	0·580
Education level						
Low	Reference					
Medium	1·44	–2·03, 4·90	0·416	−3·85	–7·85, 0·14	0·059
High	0·99	–2·83, 4·81	0·611	−2·68	–6·97, 1·61	0·220
Region						
North	Reference					
Centre	4·39	2·83, 5·94	< 0·001	7·55	4·69, 10·42	< 0·001
Mexico City	1·70	–0·34, –0·34	0·103	*		*
South	5·66	3·85, 3·85	< 0·001	9·43	6·41, 12·45	< 0·001
Ethnicity (%)						
Indigenous	Reference					
Non-indigenous	−5·35	–8·43, –2·27	< 0·001	−3·82	–6·74, –0·91	0·010
Constant	49·6	43·9, 55·2	< 0·001	60·52	54·71, 66·34	< 0·001

HEI-2015, Healthy Eating Index-2015.

Estimations based on a multiple linear regression model adjusted for the survey design and all the variables in this table.

* Mexico City is only considered as urban, with no rural areas.

**Fig. 2:**
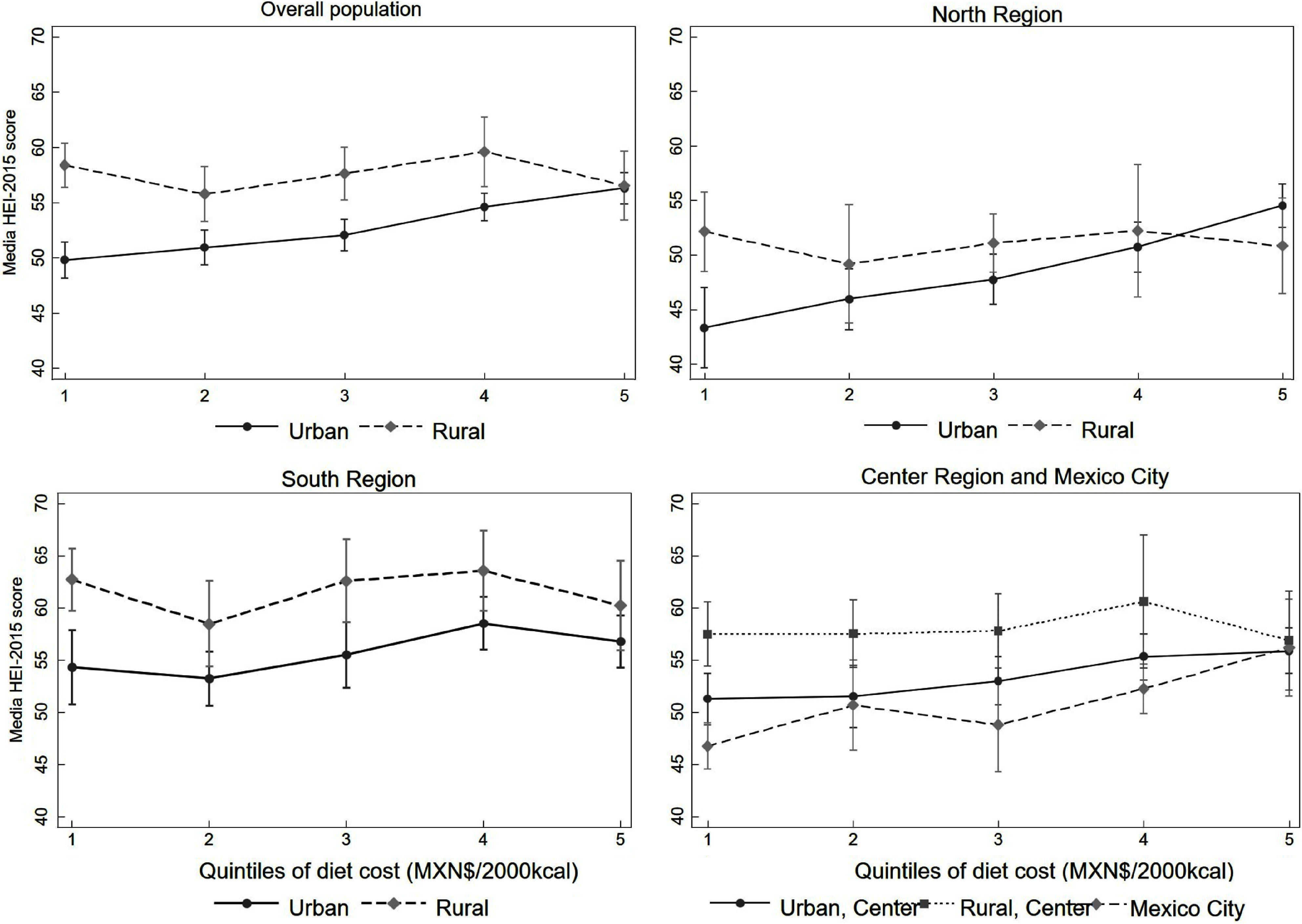
Association between diet cost quintiles and Healthy Eating Index-2015 (HEI-2015) in adults by area and region of residence – ENSANUT 2012. Predicted means of HEI-2015 score by quintiles of diet cost stratified by area and region of residence, estimated from linear regression models adjusted for sex, age, socio-economic status, education and ethnicity. In the figure of the overall population, the model includes the region; for the rest of the regions, we included the area of residence except for Mexico City, which only have urban population

As can be seen in Fig. 2, when stratifying the analysis by region and area of residence, we found that the positive association between diet cost and quality was stronger in urban areas of the North, Mexico City and the Centre region, as shown by the large difference in the values between quintiles of diet cost. Differences in the score of diet quality between the highest *v*. the lowest cost quintiles were 11·06 (95 % CI 6·78, 15·35), 7·91 (95 % CI 2·97, 12·84) and 5·20 (95 % CI 1·94, 8·45) points, respectively, while in the Southern region, the difference was 2·56 (95 % CI 1·54, 6·66) (see online supplementary material, Supplemental Table 3). No statistically significant associations were seen in rural areas of any region.

Having a medium *v*. low SES was associated with a lower diet quality in urban areas, while in rural areas having a high *v*. low SES was associated with lower diet quality, maintaining the constant average cost of diet (Table 3). The highest quintiles of cost had on average a higher HEI-2015 score in all levels of SES in urban but not in the rural areas (see online supplementary material, Supplemental Fig. 2).

Additionally, both in urban and rural areas, being indigenous compared with non-indigenous was associated with a higher diet quality. No associations were found between sex and education with diet quality (Table 3).

The sensitivity analysis using prices from INEGI-2012 showed a similar trend in the relation between diet quality and diet cost in urban and rural areas; however, the coefficients in urban areas were attenuated (see online supplementary material, Supplemental Table 4). When adjusting by energy intake, we found similar results in rural areas (see online supplementary material, Supplemental Tables 6 and 8), but in urban areas, we found larger association coefficients (see online supplementary material, Supplemental Tables 5 and S).

## Discussion

We found that the association between diet cost and diet quality differed by urban/rural area and region in Mexico. No association between diet cost and quality was observed in rural areas, while in urban areas, there was a positive association with stronger magnitude in the North, Centre and Mexico City compared with the Southern region. Our results highlight the existence of a broad overlap in the distribution of diet quality across levels of diet cost, indicating that it is possible to have a high-quality diet without increasing its cost. Also, adults living in rural *v*. urban areas, in the South *v*. Northern region, those with low *v*. high SES and indigenous *v*. non-indigenous had a higher quality diet.

To our knowledge, this is the first study that analysed the association between diet cost and overall diet quality in Mexico using a HEI. A previous national study from Mendoza *et al*. analysed this association using energy density as an indicator of diet quality and found that diets with lower energy density were associated with a higher diet cost with no differences by place of residence^(21)^. We also found that higher quality diets cost more than lower quality ones, but only in urban areas. One difference with our study is that, in contrast to the HEI-2015, energy density is not a measure of overall diet quality, since it measures only one characteristic of the diet without considering recommendations regarding other dietary components^(40)^. In comparison with international data, studies that come mainly from high-income countries reported the same association we observed in urban areas, regardless of the metric used to assess diet quality^(6)^. Also, population-based studies using a method similar to the HEI-2015 reported that higher diet cost was associated with higher quality diets^(10,11,41,42)^. However, as we reported here, a study conducted in adult females from the USA highlighted that diet quality could be improved without increasing the diet cost^(11)^. We also found that the association between diet cost and diet quality in urban areas varies by region, showing a stronger association in the most urbanised and wealthier regions (North and Mexico City), compared with the South, which is the poorest region of the country. In contrast, the studies from the USA that mostly analysed urbanised context did not report any differences by states, instead it reported a strongest positive relation of diet quality and diet cost by sex^(10,11,41,42)^.

Our findings also showed no significant association between diet cost and diet quality in rural areas, which could be due to less variability in food prices in comparison to urban areas or due to a greater measurement error in prices in rural areas using the ENIGH. However, in a sensitivity analysis using INEGI’s prices from the country’s major cities, the same source of prices as Mendoza *et al.* ^(21)^, our results did not change. At the international level, few studies have analysed the relationship between diet cost and diet quality in rural areas. One study from the rural Victoria (Australia) reported that diets were unhealthy and cost more than healthier diets^(8)^, while in poor rural areas of the USA and Australia, it was reported a low access to affordable healthy options^(43–45)^. In contrast, our study showed that even though diet quality in rural areas did not reach an optimal score (HEI > 80), rural populations had a higher diet quality at a lower cost compared with urban areas, which is explained by differences in the composition of the diet between urban and rural areas. Compared to urban areas, adults from rural areas consume not only more whole grains (mainly from maize), beans and less dairy products and animal proteins but also less sugar, Na and saturated fats that are major components of highly processed foods. This is consistent with Mendoza *et al*. who reported that rural adults in Mexico eat more traditional and low-cost energy-dense foods^(21)^.

In addition, having diets with a higher cost does not necessarily imply a higher quality score in all dietary components. We found that adults with more expensive diets, as well as those with higher SES, have not only a greater consumption of healthy components of the HEI-2015 such as fruits and whole fruits, vegetables, dairy products, animal proteins, seafood and nuts, but also a greater consumption of unhealthy dietary components such as Na, added sugar, saturated fats, and a lower ratio of unsaturated fatty acids to SFA. Another study in Mexico also found that individuals with high SES and those living in urban areas have a greater consumption of not only fruits, legumes and dairy products but also products high in sugar and saturated fat^(14)^ which mainly come from processed and ultra-processed products^(46)^. In contrast, in high-income countries such as the USA, the higher quality diets had higher adherence to most of the dietary components, except for Na intake, which could be related to their higher consumption of processed food^(10)^.

The relation between outcomes and SES was complex. In urban areas, more expensive diets showed better quality at all levels of SES, but in rural areas, for indigenous groups and those living in the South (all low-income groups), a higher diet quality was associated with the same or even lower cost. These relationships could be partly explained by differences in the effect of prices and income on food consumption. Previous studies in Mexico have shown that nutrient-rich foods such as fruits, vegetables and nuts are more expensive and have become less affordable than unhealthy options such as ultra-processed products^(20,21)^, so individuals with a greater consumption of nutrient-dense foods will have a more expensive diet. However, income is also a driver of food choice, and in a previous study in Mexico, products high in sugar, fat and Na, as well as fruits, animal protein and dairy products, were income elastic (> 1); thus, individuals with a higher income had greater consumption of these products^(47)^.

Differences with the results in other countries as well as the mixed results by area and region of residence we presented in this study suggest that the association between diet cost and diet quality as well as other related factors is country specific and that there is heterogeneity even within a country. This heterogeneity could be explained by differences in food availability, physical and economic access to food affected by the geographical conditions, as well as individual factors such as socio-economic and cultural conditions that shape food patterns as well as the different stages of nutritional transition^(15,48–50)^. This is particularly important in middle-income countries such as Mexico where this heterogeneity in the drivers of food choice may affect the effectiveness of strategies to promote better quality diets^(42)^.

In relation to the above, in Mexico, the rural and the South sub-populations live in the poorest socio-economic conditions and had a higher proportion of indigenous population in the country^(51)^. In contrast, the urban areas and the North have similar characteristics of more urbanised countries and have a higher income than other regions^(51)^. The differences in diet quality by socio-demographic conditions show the different stages of the nutritional transition within the country where urbanisation and economic growth were associated with some negative changes in dietary patterns that lead to a higher prevalence of obesity, non-communicable diseases and the coexistence with nutritional deficiencies^(48,52)^. For instance, diets of adults in urban areas and the North region are in a higher stage of the nutritional transition in comparison with diets of adults in rural areas and the South, which preserve characteristics of traditional and healthier diets such as higher consumption of whole grains, vegetables and beans and a lower intake of Na, added sugar and saturated fats. This is consistent with previous studies that show differences in diet quality, as well as the heterogeneous process of nutritional transition that is linked with a higher prevalence of obesity in the wealthier and more urbanised regions^(48,53)^. However, the influence of cultural factors associated with the place of residence and ethnicity and how they impact the quality of diet requires further analysis^(54,55)^. Additionally, the difference in diet quality and diet cost can also be influenced by the characteristics of the food supply. The food production in Mexico mainly takes place in rural areas, so families could have access to their own food production. Also, in the Northern region, the supermarkets are the main supply of food, while in the Southern region, the open markets are more present. In all the regions, grocery stores are major food suppliers for households^(51)^. This heterogeneity in the food supply is also associated with different food prices and food quality^(52)^. We can hypothesise that this heterogeneity provides the possibility of improving diet quality at the same or even lower cost compared with urban areas. Additionally, a study of food environments in Mexico showed that in rural areas, there is access to healthy food options, but there is greater access and exposure to unhealthy options^(53)^. This is different in the rural areas of the USA where a limited access to affordable healthy food was identified as a main problem^(54)^. However, unless any action is taken to improve the consumption of healthier options, the dietary patterns of rural areas could worsen as economic conditions improve and unhealthy products become more affordable^(20)^.

In addition, it is interesting to note that low SES and being indigenous seem to protect the adults from adopting lower quality diets, mainly due to their history of undernutrition. However, it is important to note that on average, the diet quality in the adult Mexican population was suboptimal (54·1 out of 100 points), even in those sub-populations with higher diet quality, such as indigenous population, as well as those from the South and rural areas (62·2, 57·7 and 59·7 points, respectively). So, those sub-populations have relatively higher diet quality, but this is still not optimal. This could explain the persistence of the current nutritional problems such as micronutrient deficiencies, especially the low intake of bioavailable Fe and vitamin B_12_^(55)^, and overweight/obesity which is on a rapid increase^(56)^. Furthermore, our results of diet quality are determined by the HEI-2015 which assessed the overall diet based on the adherence to the recommendation of the Dietary Guidelines for Americans considering both adequacy and moderation components that are related to the prevention of nutritional deficiencies, overweight and non-communicable diseases. These recommendations are in line with the latest international ones considered also in the Mexican dietary guidelines^(57)^. Also studies that adapted the HEI-2015 to the Mexican dietary recommendation showed also a suboptimal diet quality with the same association with socio-demographic factors (higher diet quality in rural areas, the South region and those with low SES)^(16,58)^. Similar results were reported using other methods to assess the diet quality such as *a posteriori* dietary patterns and by food groups^(14,15,59)^. This suggest that the use of the HEI-2015 gives consistent results of diet quality in the context of Mexican adult population considering their major health problems.

This study has some limitations and strengths. We derived the food prices from a national expenditure survey (ENIGH 2012), which did not collect the food prices directly. This may have introduced measurement error; however, prices were estimated at the municipality level to reduce bias at the household level. Furthermore, in the estimation of food prices, we included the monetary values of food that was reported as home-produced food. Also, a sensitivity analysis using food prices directly collected by the INEGI in urban cities did not change our results. In addition, the SFFQ includes a limited list of foods and thus could lead to underestimating food intake. However, these foods represented more than 90 % of the total energy and nutrient intake and are a measure of habitual intake. Likewise, the amount of Na intake is underestimated because we did not include the salt added to food. As those errors affect all of the study population, and the cost of salt is minimal, we do not expect any differential bias. There is also a potential response bias for a differential misreporting of the amount of food consumed by place of residence that could lead to under or overestimation of the price as well as the HEI-2015. The stratified analysis by place of residence and the adjustment for energy and socio-economic covariates may reduce these effects. Despite those limitations, this is the first study that analysed the association between diet cost and overall diet quality in a representative sample of the Mexican adult population that could be used as a baseline for future research in this field. Also, the use of standardised methods reduced the potential selection bias and measurement errors, and we included sensitivity analyses to test the consistency of our results.

### Policy implications and future research

Our results revealed some potential practical implications. Further studies are needed to better understand the relation of diet cost and diet quality, as well as the main drivers of diet quality in rural areas and regions, and between levels of SES in Mexico. A deeper analysis of the diets as well as the strategies and conditions that allow some sub-populations to have higher quality diets at a lower cost could provide useful information for the design of interventions to promote better diets. Continuation of policies to reduce the consumption of nutritionally poor, processed foods and sugary beverages, such as taxes, front of pack labelling and regulations to the school’s food environment, complemented with policies to increase the consumption of healthy food groups, such as subsidies to fruits and vegetables in urban areas to make them more affordable and the implementation of healthy and sustainable food-based dietary recommendations for Mexican population^(52)^ should consider the heterogeneity in the drivers of diet quality by place of residence. Also, strengthening the population’s capacity to make better food choices considering their cultural characteristics could improve the effectiveness of policies aiming to promote better quality diets in Mexico.

## Conclusions

Diet cost was positively associated with diet quality, but only in urban areas, with the strongest association in the North, Mexico City and the Central region. Being indigenous, having a low SES, living in the South and in rural areas were also associated with higher quality diets. Current food policies in Mexico could prove more effective by including elements to improve the consumption of healthy food groups that consider the heterogeneity in the drivers of diet quality by place of residence.

## Supplementary material

For supplementary material accompanying this paper visit https://doi.org/10.1017/S1368980021004651. click here to view supplementary material
