# Supplementary material for: Diet cost and quality using the Healthy Eating Index-2015 in adults from urban and rural areas of Mexico
Source: Public Health Nutr. 2021 Nov 24;25(9):2554–65. doi: 10.1017/S1368980021004651 (PMC9991559; doi:10.1017/S1368980021004651)
Supplement: Supplementary file 1 [file S1368980021004651sup.zip › S1368980021004651sup001.docx]

**Diet cost and quality using the Healthy Eating Index-2015 in adults from urban and rural areas of Mexico**

Online Supplementary Material

Table S1. Dietary components and scoring criteria for construction of HEI-2015^1^

| **Dietary component** | **Maximum points** | **Standard for maximum score*** | **Standard for minimum score of zero** |
| --- | --- | --- | --- |
| **Adequacy:** |  |  |  |
| Total Fruits ^2^ | 5 | ≥0.8 cup equiv. per 1,000 kcal | No Fruit |
| Whole Fruits ^3^ | 5 | ≥0.4 cup equiv. per 1,000 kcal | No whole Fruit |
| Total Vegetables ^4*^ | 5 | ≥1.1 cup equiv. per 1,000 kcal | No vegetables |
| Greens and Beans ^5*^ | 5 | ≥0.2 cup equiv. per 1,000 kcal | No dark green vegetables or legumes |
| Whole Grains | 10 | ≥1.5 oz equiv. per 1,000 kcal | No whole grains |
| Dairy ^6^ | 10 | ≥1.3 cup equiv. per 1,000 kcal | No dairy |
| Total Protein Foods ^7*^ | 5 | ≥2.5 oz equiv. per 1,000 kcal | No protein Foods |
| Seafood and Plant Proteins ^8*^ | 5 | ≥0.8 oz equiv. per 1,000 kcal | No seafood or plant proteins |
| Fatty Acids ^9^ | 10 | (PUFAs + MUFAs)/SFAs ≥2.5 | (PUFAs + MUFAs)/SFAs ≤1.2 |
| **Moderation:** |  |  |  |
| Refined Grains | 10 | ≤1.8 oz equiv. per 1,000 kcal | ≥4.3 oz equiv. per 1,000 kcal |
| Sodium | 10 | ≤1.1 gram per 1,000 kcal | ≥2.0 grams per 1,000 kcal |
| Added Sugars | 10 | ≤6.5% of energy | ≥26% of energy |
| Saturated Fats | 10 | ≤8% of energy | ≥16% of energy |
| *The units for each dietary component are called unit equivalents, for example the unit equivalent for total fruits is the cup equivalent per 1000 kcal, for whole grains is the ounce equivalent per 1000 kcal, etc. | | | |

## Intakes between the minimum and maximum standards are scored proportionately.

## Includes 100% fruit juice.

## Includes all forms except juice.

## *Originally includes legumes (beans and peas). For this study we included only vegetables and fresh green peas.

## *Originally includes legumes (beans and peas). For this study we included only legumes (beans, lentils, chickpeas, etc.)

## Includes all milk products, such as fluid milk, yogurt, and cheese, and fortified soy beverages.

## *Originally includes legumes (beans and peas). For this study we included only protein from animal sources.

## *Originally includes seafood, nuts, seeds, soy products (other than beverages), and legumes (beans and peas). For this study we excluded soy products and legumes.

## Ratio of poly- and monounsaturated fatty acids (PUFAs and MUFAs) to saturated fatty acids (SFAs).

*Based on NCI. Developing the Healthy Eating index. Available from https://epi.grants.cancer.gov/hei/developing.html*

Figure S1. Flow chart of the analytical sample.

Sub-sample with SFFQ data from ENSANUT-2012 n=7810*

Adults from 18 to 59 y.

n=2792

Excluded: Pregnant or lactating women n=147

Excluded: Implausible nutrient intake values n= 207

Analytical sample of adults from 18 to 59 y. n=2438

*Includes population from different age-groups (from pre-school children to older adults).

*Based on Ramirez et al 2016. Methodology for estimating dietary data from the semi quantitative food frequency questionnaire (SFFQ) of the Mexican National Health and Nutrition Survey 2012 (ENSANUT-2012).*

Table S2. Association between diet cost quintiles and HEI-2015 in models with the inclusion of increasing numbers of covariates.

|  | **HEI-2015 score Adjusted Coefficient (95% CI)** | | **P-value** |
| --- | --- | --- | --- |
| **Model 1: Crude (Diet cost and HEI-2015)** | | | |
| Quintiles of diet cost (MXN$/2000kcal) |  |  |  |
| Q1 | Reference |  |  |
| Q2 | -2.56 | (-4.69;-0.44) | 0.018 |
| Q3 | -2.23 | (-4.28;-0.17) | 0.034 |
| Q4 | -0.32 | (-2.34;1.70) | 0.758 |
| Q5 | 0.93 | (-1.01;2.87) | 0.348 |
| **Model 2: Model 1 + age, sex, SES, education and ethnicity** | | | |
|  | **HEI-2015 score Adjusted Coefficient (95% CI)** | | **P-value** |
| Quintiles of diet cost (MXN$/2000kcal) |  |  |  |
| Q1 | Reference |  |  |
| Q2 | -1.10 | (-2.99;0.79) | 0.254 |
| Q3 | -0.15 | (-2.13;1.83) | 0.881 |
| Q4 | 1.94 | (0.01;3.87) | 0.049 |
| Q5 | 3.23 | (1.27;5.19) | 0.001 |
| **Model 3: Model 2 +Interaction area and diet cost quintiles + region** | | | |
|  | **HEI-2015 score Adjusted Coefficient (95% CI)** | | **P-value** |
| Quintiles of diet cost (MXN$/2000kcal) |  |  |  |
| Q1 | Reference |  |  |
| Q2 | 1.15 | (-1.07;3.36) | 0.309 |
| Q3 | 2.27 | (0.05;4.48) | 0.045 |
| Q4 | 4.80 | (2.77;6.83) | <0.001 |
| Q5 | 6.53 | (4.37;8.70) | <0.001 |
| Area of residence | 8.59 | (5.97;11.22) | <0.001 |
| Quintiles of diet cost x area of residence |  |  |  |
| Q2 x Rural | -3.76 | (-7.54;0.17) | 0.051 |
| Q3 x Rural | -3.03 | (-6.72;0.66) | 0.107 |
| Q4 x Rural | -3.58 | (-7.89;0.72) | 0.103 |
| Q5 x Rural | -8.37 | (-12.58;-4.17) | <0.001 |

**Figure S2.** Diet quality* (HEI-2015) by quintiles of diet cost by socioeconomic status and area of residence

*Diet quality was adjusted for sex, age, socioeconomic status, education, and ethnicity using the multiple linear regression model.

Table S3. Association between diet cost quintiles and HEI-2015 among regions

| **Quintiles of diet cost (MXN$/2000 kcal)** | **HEI-2015 score ^a^ Adjusted Coefficient (95% CI)** | | **P-value** |
| --- | --- | --- | --- |
| **North Region (Mean+SD)** |  |  |  |
| Q1 | Reference |  |  |
| Q2 | 2.76 | (-1.72 , 7.24) | 0.226 |
| Q3 | 4.40 | (0.52 , 8.29) | 0.027 |
| Q4 | 7.38 | (3.12 , 11.63) | <0.001 |
| Q5 | 11.06 | (6.78 , 15.35) | <0.001 |
| **Mexico City (Mean+SD)** |  |  |  |
| Q1 | Reference |  |  |
| Q2 | 3.20 | (-1.63 , 8.02) | 0.190 |
| Q3 | 0.42 | (-4.58 , 5.42) | 0.867 |
| Q4 | 4.16 | (0.58 , 7.74) | <0.001 |
| Q5 | 7.91 | (2.97 , 12.84) | <0.001 |
| **Center (Mean+SD)** |  |  |  |
| Q1 | Reference |  |  |
| Q2 | 0.74 | (-3.16 , 4.64) | 0.708 |
| Q3 | 2.17 | (-1.35 , 5.68) | 0.227 |
| Q4 | 4.83 | (1.53 , 8.13) | 0.004 |
| Q5 | 5.20 | (1.94 , 8.45) | 0.002 |
| **South Region (Mean+SD)** |  |  |  |
| Q1 | Reference |  |  |
| Q2 | -1.04 | (-5.49 , 3.42) | 0.648 |
| Q3 | 1.27 | (-3.50 , 6.03) | 0.602 |
| Q4 | 4.47 | (0.31 , 8.64) | 0.035 |
| Q5 | 2.56 | (-1.54 , 6.66) | 0.221 |

Estimations based on multiple linear regression model adjusted for age, sex, ethnicity, education, socioeconomic status and the term of interaction of diet cost and area of residence.

Table S4. Association between diet cost and HEI-2015 in urban/rural, using prices of INEGI-2012.

| **Quintiles of diet cost (MXN$/2000kcal)** | **HEI-2015 score ^a^ Adjusted Coefficient (95% CI)** | | **P-value** |
| --- | --- | --- | --- |
| **Urban (Mean+SD)** |  |  |  |
| Q1 | Reference |  |  |
| Q2 | -0.32 | (-2.48 , 1.84) | 0.770 |
| Q3 | 0.04 | (-2.21 , 2.29) | 0.974 |
| Q4 | 3.18 | (0.95 , 5.40) | 0.005 |
| Q5 | 4.09 | (1.91 , 6.28) | <0.001 |
| **Rural areas (Mean+SD)** |  |  |  |
| Q1 | Reference |  |  |
| Q2 | -2.46 | (-5.81 , 0.89) | 0.150 |
| Q3 | -2.08 | (-5.06 , 0.90) | 0.171 |
| Q4 | -1.36 | (-4.59 , 1.87) | 0.409 |
| Q5 | -0.54 | (-4.16 , 3.09) | 0.772 |

^a^ Coefficients predicted from a regression model adjusted for sex, age, socioeconomic status, education, ethnicity and region. *Diet cost quintiles is adjusted to 2000 kcal.

Table S.5. Association between diet cost and HEI-2015 in urban/rural, using prices of INEGI-2012.

The model is adjusted by total energy intake.

| **Quintiles of diet cost (MXN$)** | **HEI-2015 score ^a^ Adjusted Coefficient (95% CI)** | | **P-value** |
| --- | --- | --- | --- |
| **Urban (Mean+SD)** |  |  |  |
| Q1 | Reference |  |  |
| Q2 | 3.14 | (0.79 , 5.49) | 0.009 |
| Q3 | 3.56 | (1.35 , 5.78) | <0.001 |
| Q4 | 5.01 | (2.46 , 7.55) | 0.001 |
| Q5 | 8.14 | (4.99 , 11.28) | <0.001 |
| **Rural areas (Mean+SD)** |  |  |  |
| Q1 | Reference |  |  |
| Q2 | -0.12 | (-3.53 , 3.30) | 0.946 |
| Q3 | -2.69 | (-6.89 , 1.51) | 0.209 |
| Q4 | -1.03 | (-5.39 , 3.32) | 0.641 |
| Q5 | 1.71 | (-3.93 , 7.34) | 0.552 |

^a^ Coefficients predicted from a regression model adjusted for sex, age, socioeconomic status, education, ethnicity, region and total energy intake. *Diet cost quintiles are not energy adjusted. The diet cost was derived from INEGI-2012 food prices database that are representative of urban areas.

Table S6. Association between diet cost and HEI-2015 in urban/rural, using prices of INEGI-2012.

The model is not energy adjusted.

| **Quintiles of diet cost (MXN$)*** | **HEI-2015 score ^a^ Adjusted Coefficient (95% CI)** | | **P-value** |
| --- | --- | --- | --- |
| **Urban area** |  |  |  |
| Q1 | Reference |  |  |
| Q2 | 2.51 | (0.20 , 4.82) | 0.033 |
| Q3 | 2.27 | (0.22 , 4.32) | 0.030 |
| Q4 | 2.95 | (0.81 , 5.10) | 0.007 |
| Q5 | 4.53 | (2.33 , 6.74) | <0.001 |
| **Rural area** |  |  |  |
| Q1 | Reference |  |  |
| Q2 | 0.04 | (-3.12 , 3.20) | 0.980 |
| Q3 | -2.39 | (-6.04 , 1.25) | 0.197 |
| Q4 | -0.60 | (-4.19 , 2.98) | 0.742 |
| Q5 | 2.34 | (-1.55 , 6.24) | 0.237 |

^a^ Coefficients predicted from a regression model adjusted for sex, age, socioeconomic status, education, ethnicity, and region. *Diet cost quintiles are not energy adjusted. The diet cost was derived from INEGI-2012 food prices database that are representative of urban areas.

Table S7 Association between diet cost and HEI-2015 in urban and rural areas. The model is adjusted by total energy intake.

| **Quintiles of diet cost (MXN$)*** | **HEI-2015 score ^a^ Adjusted Coefficient (95% CI)** | | **P-value** |
| --- | --- | --- | --- |
| **Urban área** |  |  |  |
| Q1 | Reference |  |  |
| Q2 | 3.39 | (1.09 , 5.69) | 0.004 |
| Q3 | 5.47 | (3.09 , 7.85) | <0.001 |
| Q4 | 6.70 | (4.08 , 9.32) | <0.001 |
| Q5 | 10.41 | (7.33 , 13.49) | <0.001 |
| **Rural area** |  |  |  |
| Q1 | Reference |  |  |
| Q2 | -0.64 | (-4.27 , 2.99) | 0.728 |
| Q3 | -2.83 | (-6.36 , 0.71) | 0.116 |
| Q4 | 0.37 | (-4.20 , 4.94) | 0.874 |
| Q5 | 1.71 | (-3.59 , 7.01) | 0.525 |

^a^ Coefficients predicted from a regression model adjusted for sex, age, socioeconomic status, education, ethnicity, region and total energy intake. *Diet cost quintiles are not energy adjusted.

Table S8. Association between diet cost and HEI-2015 in urban and rural areas. The model is not energy adjusted.

| **Quintiles of diet cost (MXN$)*** | **HEI-2015 score ^a^ Adjusted Coefficient (95% CI)** | | **P-value** |
| --- | --- | --- | --- |
| **Urban area** |  |  |  |
| Q1 | Reference |  |  |
| Q2 | 2.50 | (0.27 , 4.74) | 0.028 |
| Q3 | 3.87 | (1.65 , 6.10) | 0.001 |
| Q4 | 3.77 | (1.45 , 6.10) | 0.002 |
| Q5 | 5.69 | (3.50 , 7.88) | <0.001 |
| **Rural area** |  |  |  |
| Q1 | Reference |  |  |
| Q2 | -0.51 | (-3.88 , 2.86) | 0.765 |
| Q3 | -2.58 | (-5.61 , 0.44) | 0.094 |
| Q4 | 0.69 | (-3.37 , 4.74) | 0.739 |
| Q5 | 2.24 | (-1.36 , 5.83) | 0.222 |

^a^ Coefficients predicted from a regression model adjusted for sex, age, socioeconomic status, education, ethnicity and region. *Diet cost quintiles are not energy adjusted.
